# Generating cause of death information to inform health policy: implementation of an automated verbal autopsy system in the Solomon Islands

**DOI:** 10.1186/s12889-021-12180-y

**Published:** 2021-11-13

**Authors:** Matthew Reeve, Hafizur Chowdhury, Pasyodun Koralage Buddhika Mahesh, Gregory Jilini, Rooney Jagilly, Baakai Kamoriki, Rodley Ruskin, Deirdre McLaughlin, Alan D. Lopez

**Affiliations:** 1 Bloomberg Philanthropies Data for Health Initiative, University of Melbourne, Melbourne, Australia; 2 Ministry of Health & Medical Services, Honiara, Solomon Islands; 3 University of Washington, Seattle, USA

**Keywords:** Verbal autopsy, Solomon Islands, Civil registration and vital statistics, SmartVA, Tariff method, Causes of death

## Abstract

**Background:**

Good quality cause of death (COD) information is fundamental for formulating and evaluating public health policy; yet most deaths in developing countries, including the Solomon Islands, occur at home without medical certification of cause of death (MCCOD). As a result, COD data in such contexts are often of limited use for policy and planning. Verbal autopsies (VAs) are a cost-effective way of generating reliable COD information in populations lacking comprehensive MCCOD coverage, but this method has not previously been applied in the Solomon Islands. This study describes the establishment of a VA system to estimate the cause specific mortality fractions (CSMFs) for community deaths that are not medically certified in the Solomon Islands.

**Methods:**

Automated VA methods (SmartVA) were introduced into the Solomon Islands in 2016. Trained data collectors (nurses) conducted VAs on eligible deaths to December 2020 using electronic tablet devices and VA responses were analysed using the Tariff 2.0 automated diagnostic algorithm. CSMFs were generated for both non-inpatient deaths in hospitals (i.e. ‘dead on/by arrival’) and community deaths.

**Results:**

VA was applied to 914 adolescent-and-adult deaths with a median (IQR) age of 62 (45–75) years, 61% of whom were males. A specific COD could be diagnosed for more than 85% of deaths. The leading causes of death for both sexes combined were: ischemic heart disease (16.3%), stroke (13.5%), diabetes (8.1%), pneumonia (5.7%) and chronic-respiratory disease (4.8%). Stroke was the top-ranked cause for females, and ischaemic heart disease the leading cause for males. The CSMFs from the VAs were similar to Global Burden of Disease (GBD) estimates. Overall, non-communicable diseases (NCDs) accounted for 73% of adult deaths; communicable, maternal and nutritional conditions 15%, and injuries 12%. Six of the ten leading causes reported for facility deaths in the Solomon Islands were also identified as leading causes of community deaths based on the VA diagnoses.

**Conclusions:**

NCDs are the leading cause of adult deaths in the Solomon Islands. Automated VA methods are an effective means of generating reliable COD information for community deaths in the Solomon Islands and should be routinely incorporated into the national mortality surveillance system.

**Supplementary Information:**

The online version contains supplementary material available at 10.1186/s12889-021-12180-y.

## Background

Good quality mortality statistics, especially on underlying causes of death, are essential for effective health system planning, management and evaluation [1, 2] and are a critical element of a country’s civil registration and vital statistics (CRVS) system. Enabling countries to generate and use reliable mortality data has, therefore, increasingly been seen as a global health priority [3]. Most deaths in resource-constrained countries occur in community settings, often not certified by a trained medical practitioner [4, 5]. Physician certification of cause of death (COD) is the ‘gold standard’ for generating mortality data to guide policy and planning in the health sector. However, if these data are only available for deaths in health facilities, the resulting COD data are of limited use for understanding mortality trends in the population as a whole, particularly in communities with limited access to health facilities.

The Solomon Islands is a Pacific island country comprised of nearly 1000 islands with a projected population of nearly 700,000 in 2020 [6]. The crude death rate as estimated by the 2009 census was 5.5 per 1000 population [7]. There is evidence that at least some mortality indicators (e.g. neonatal, under five mortality) vary across certain geographical settings (i.e. urban and rural) [7, 8]. The mortality reporting systems of Pacific island countries in general have been identified as facing many societal, administrative and system related challenges [9]. The limited available information on mortality patterns in the Solomon Islands is almost exclusively from hospital settings [10, 11]; very little has been published on the causes of the majority of deaths, where physician certification was not possible.

The Solomon Islands has ten hospitals with physicians, but a substantial proportion of the population in remote or rural settings have limited access to hospital services, and therefore, to physician certification of death. About 75% of deaths reported in the Solomon Islands do not receive a medical certification of cause of death (MCCOD) [12]. Health facilities outside hospitals are staffed exclusively by nurses, nurse aides or allied health practitioners who are not trained or legally qualified to certify deaths. Nurses in primary health facilities are responsible for notifying the Ministry of Health and Medical Services with demographic details of any deaths in their catchment area, but these data are very incomplete and do not contain information about the COD. Little is therefore known about mortality patterns in rural and remote areas beyond any hospital deaths of people referred from these settings; and representative national COD data has not been generated [10]. This key data gap results in public policy which depends heavily on projections from hospital mortality data, or international estimates such as the Global Burden of Disease (GBD) study, whose modelling for Solomon Islands is based on mortality patterns in neighbouring countries in the absence of local COD data.

To fill this key gap in the national health information system, the SmartVA automated verbal autopsy (VA) methodology was introduced into the Solomon Islands under the Bloomberg Philanthropies Data for Health (D4H) Initiative in 2016, in accordance with the unmet priority objectives from the National CRVS Improvement Plan [13]. VAs are interviews conducted with an informant familiar with the medical history of the deceased, particularly the signs and symptoms preceding death. These interview data are then interpreted by either a physician, or computer diagnostic algorithms, and assigned a most likely underlying COD [14–16].

In this paper we present findings from the first ever application of an automated verbal autopsy system, to determine the leading causes of community deaths in Solomon Islands, thus filling a key knowledge gap for health policy and planning in the country. We describe the application of the SmartVA automated diagnostic method and discuss the potential policy utility of the SmartVA methodology for routinely generating evidence about the causes of community deaths in the Solomon Islands.

## Methods

A National Mortality Technical Working Group (NMTWG) was established with representation from the Ministry of Health and Medical Services, and the country office of the World Health Organization (WHO) to oversee rollout of the VA system within government health facilities. The system was piloted in Guadalcanal Province, Western Province, and Honiara capital district (separately administered, but located on Guadalcanal island) in 2016, and progressively scaled up to national coverage by 2018. In each province, data collection took place in hospital emergency departments (for those deaths on or soon after arrival that could not be certified by a physician), and for community deaths reported to health facilities below hospital level.

### Training and data collection

Data were collected on Android tablets using the Population Health Metrics and Research Consortium (PHMRC) shortened questionnaire, implemented using the Open Data Kit (ODK) suite of software [17]. The Smart VA questionnaire has three age-specific modules: neonatal deaths (from birth to 28 days), child deaths (from 29 days up to 11 years), and adult deaths (from 12 years onwards) [16, 18].

Data were collected by nurses working in hospital emergency departments and Area Health Centres (AHCs, the facility level below hospitals). Nurses underwent a five-day training covering the importance of quality CRVS data, the questionnaire, hardware and software management, interview techniques, interview ethics, and cultural considerations. Within this cohort, some data collectors also received additional training to enable them to act as master trainers to support the induction of new personnel to collect data. All field interviewers were issued with a digital tablet with a data-enabled SIM card, and a solar charger for locations with unreliable mains electricity supply. Nurses based at AHCs were responsible for conducting VAs on deaths which occurred in the facility’s catchment area, including while on supervisory visits to lower-level facilities. Data were uploaded directly to a secure server whenever nurses had access to adequate mobile data coverage or wi-fi. For those locations where internet access was not regularly available, field staff were given tablet-compatible USB storage devices and trained in uploading interview data onto the device, which was then sent by hand or post to the provincial capital for uploading to the server.

### Study population and sample

Any deaths which occurred within a period of twelve months prior to the date of data collection were eligible for a VA. This time period allowed for some lag in the initial notification of deaths while minimising the risk of interviewee recall bias. Deaths which had undergone an MCCOD were not eligible for VA. We distinguished two categories of deaths based on location of data collection: (1) hospital-related deaths (i.e. either deaths recorded in hospitals that occurred en route to the hospital or shortly after arrival, and therefore were not eligible for a MCCOD, or inpatient deaths in a hospital where no doctors were on staff); and (2) community deaths (home deaths and deaths that occurred in a community setting outside the home).

### Data analysis

Data from the central server were analysed using SmartVA Analyse Tariff 2.0 software. Tariff 2.0 uses an algorithm to assign a probable COD based on VA responses, developed using a gold standard set of VAs conducted on physician-certified deaths. The algorithm generates a list of 33 adult cause specific mortality fractions (CSMFs) (Supplementary Table 1) [19]. When a VA did not contain enough specific data to be assigned a probable COD, it was categorised as “undetermined”. Undetermined cases were subsequently fractionally reallocated to specific COD categories at the population level, based on the frequency of each underlying cause being assessed by the algorithm as undetermined in the gold standard database used to produce the algorithm. This redistribution was weighted according to the estimated age-sex COD distribution for Solomon Islands, based on GBD models of the relationship of causes of death with specific covariates [19].

Prior to the introduction of automated VA, there were two estimates of overall cause-specific mortality available for health planning in Solomon Islands: physician-certified (hospital based) mortality data, and modelled estimates from the GBD study. We compared our findings with both, as a general plausibility check and in order to highlight any differences which might arise from application of this new methodology applied to this specific sample of community deaths. Comparisons with physician-certified deaths were made based on the International Statistical Classification of Diseases and Related Health Problems (ICD) codes assigned as the underlying COD for hospital deaths among persons aged 15 years and over who died between January 2016 and August 2019, as close as possible a match to the age and time range of the VA data given the ICD data available to us. These ICD codes were mapped to the VA COD (Supplementary Table 1) [20]. Comparison with the GBD estimates was carried out using the Verbal Autopsy Interpretation and Performance Evaluation Resource (VIPER) tool, which facilitates analysis and comparison of COD data from VA with different sources [21].

## Results

From January 2016 to December 2020, 1034 completed VA interviews were collected. The distribution of deaths by sex and place of residence is shown in Table 1. Nearly 60% of deaths were of residents of Guadalcanal, Western Provinces and Honiara, the sites where the program was first established. More male deaths were recorded, overall and in most provinces. In 2019, the first full year with national scale coverage, an estimated 12% of community deaths in the country in that year were covered by VA.

**Table 1:** Number of VAs conducted by sex and province, Solomon Islands, 2016–20

Province	Males		Females		Total^a^	
	N	%	N	%	N	%
Guadalcanal	144	62.3	87	37.7	231	100.0
Western	180	64.1	101	35.9	281	100.0
Malaita	74	60.2	49	39.8	123	100.0
Temotu	14	56.0	11	44.0	25	100.0
Choiseul	63	58.3	45	41.7	108	100.0
Makira-Ulawa	26	50.0	25	48.1	52	100.0
Honiara	62	73.8	22	26.2	84	100.0
Rennell-Bellona	16	51.6	15	48.4	31	100.0
Central	36	55.4	29	44.6	65	100.0
Isabel	20	62.5	12	37.5	32	100.0
Unknown	1	50.0	1	50.0	2	100.0
Total	636	61.5	397	38.4	1034	100.0

^a^The sex was not recorded for one neonatal death in Makira-Ulawa

Most deaths recorded were of adolescents or, primarily, adults. As all VA systems based on the WHO guidelines classify decedents aged 12 years or older as ‘adults’, we have used the term ‘adults’ to refer to any death in this age range. Table 2 shows the age-sex characteristics of the deaths. Adult deaths comprised 88% of cases, more (61%) among males. The median age at death for adult females was 5 years older than adult males (65 years compared to 60 years). More details on the age and sex distribution of deaths, and comparisons with GBD estimates, can be found in Supplementary Table 2. While the sex ratio of VA deaths was skewed towards males, compared with the GBD estimates, the proportion of deaths in each age category was similar between the VA data and GBD estimates, including the proportion of deaths that were neonatal or child deaths.

**Table 2:** Age and sex distribution of VA deaths, Solomon Islands, 2016–20

	Male			Female			Total		
	N	%	Median age (IQR)	N	%	Median age (IQR)	N	%	Median age (IQR)
Adult (12+ yrs)	560	61.3	60 (45.0–74.0) yrs	354	38.7	65 (48.0–78.0) yrs	914	100.0	62.0 (45.8–75.0) yrs
Child (29 days – 11 yrs)	56	62.9	12 months (5–60 months)	33	37.1	12 months (6–54 months)	89	100.0	12 months (5–60 months)
Neonate (0–28 days)	20	64.5	1.0 day (0.0 to 8) days	10	32.3	0.0 days (0 to 0.3) days	31^a^	100.0	0.0 (0 to 5) days

^a^The sex was not recorded for one neonatal death

Due to the relatively small number of child and neonate deaths in our sample, we restrict our COD analyses here to adult deaths (*n* = 914) only. Of these, 23 deaths were of people aged 12–19 years.

The leading 15 CSMFs by sex, before and after re-distribution of undetermined causes, are reported in Table 3. The algorithm was able to assign a specific COD for 86.5% of adult deaths (87.7% for males and 84.7% for females), with the COD being undetermined in approximately 15% of cases. For both sexes combined, the leading five causes of death before re-distribution were: ischemic heart disease (16.3%), stroke (14.4%), diabetes (8.1%), pneumonia (5.7%) and chronic respiratory disease (4.8%). The same five causes dominated the cause of death pattern for males, while for females, stroke was the top ranked cause and cervical cancer was also among the leading five causes.

**Table 3:** Leading 15 cause-specific mortality fractions for all VA deaths, before and after re-distribution of undetermined causes, Solomon Islands, 2016–20

Cause^a^	Before re-distribution			After re-distribution		
	Male	Females	Total	Male	Females	Total
	N (%)	N (%)	N (%)	(%)	(%)	(%)
Ischemic heart disease	108 (19.3)	41 (11.6)	149 (16.3)	20.6	13.0	17.6
Stroke	87 (15.5)	45 (12.7)	132 (14.4)	16.6	14.1	15.7
Diabetes	41 (7.3)	33 (9.3)	74 (8.1)	8.0	10.4	8.9
Pneumonia	32 (5.7)	20 (5.6)	52 (5.7)	6.4	6.6	6.5
Chronic respiratory disease	24 (4.3)	20 (5.6)	44 (4.8)	5.0	6.5	5.6
Other NCDs^b^	20 (3.6)	10 (2.8)	30 (3.3)	4.5	4.0	4.3
Other injuries^c^	22 (3.9)	6 (1.7)	28 (3.1)	4.4	2.2	3.5
Cirrhosis	23 (4.1)	5 (1.4)	28 (3.1)	4.5	1.7	3.4
Falls	17 (3.0)	10 (2.8)	27 (3.0)	3.2	3.1	3.1
Cervical cancer	0 (0.0)	25 (7.1)	25 (2.7)	0.0	7.2	2.8
Leukemia/lymphomas	9 (1.6)	14 (4.0)	23 (2.5)	1.9	4.2	2.7
Malaria	14 (2.5)	9 (2.5)	23 (2.5)	3.2	3.4	3.3
Chronic kidney disease	11 (2.0)	9 (2.5)	20 (2.2)	2.4	3.3	2.8
Breast cancer	0 (0.0)	17 (4.8)	17 (1.9)	0.0	5.0	1.9
Drowning	11 (2.0)	1 (0.3)	12 (1.3)	2.2	0.5	1.6

^a^The relevant ICD codes are shown in supplementary Table 1

^b^NCDs which are not included within another specific cause category in the SmartVA cause list

^c^Injuries which are not included within another specific cause category in the SmartVA cause list

Data were also analysed by place of residence of the deceased, according to the three phased areas where VA was deployed: Guadalcanal (including Honiara capital territory), then Western Province, and finally all other provinces as the third group. The provinces where VA was deployed earlier had lower rates of undetermined deaths (Supplementary Table 3). Guadalcanal (including Honiara) and Western provinces had the highest rates of ischemic heart disease. Stroke was relatively less common as a COD in Guadalcanal island compared to Western and other provinces, while pneumonia was more common.

**Fig. 1:**
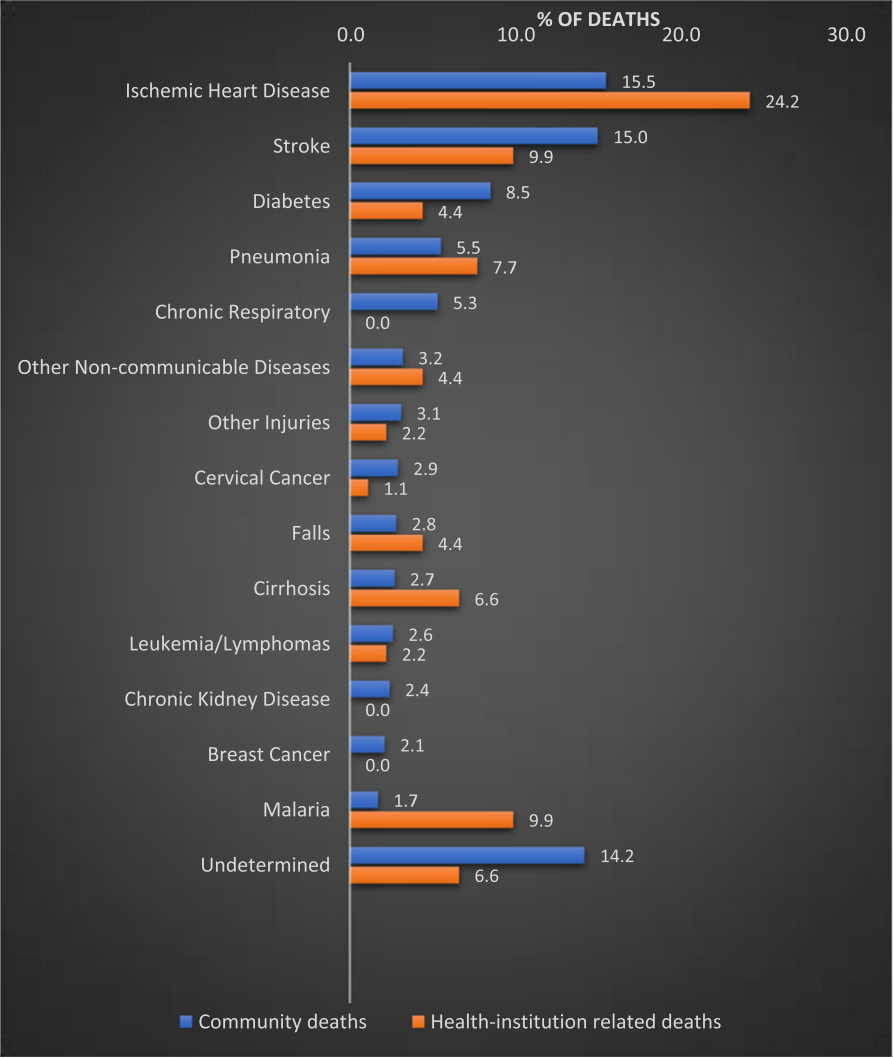
Distribution of causes of adult hospital-related deaths and community deaths from VA data, Solomon Islands, 2016–20

Figure 1 shows the comparison between CSMFs for VAs for community deaths (*n* = 647) and CSMFs for hospital-related deaths (*n* = 83). The location of three adult deaths was unspecified and they were excluded from this analysis. Community deaths typically occurred at more advanced ages, with a median age at death of 63 years (IQR: 48–77), compared to 50 years (IQR: 36–65) for those who died in hospital (Mann Whitney U test *p* < 0.001 for difference in age distribution). The male to female ratio among community deaths was 60:40, lower than among hospital-related deaths (69:31). Stroke was the leading cause among community deaths, while ischemic heart disease was the leading cause of hospital deaths. Deaths due to ischemic heart disease, pneumonia, road traffic accidents, falls, malaria and cirrhosis were higher among hospital related deaths. Conversely, diabetes, stroke, chronic respiratory and chronic kidney conditions were more common as causes of community deaths. The proportion of deaths with an undetermined cause, often an indicator of the general quality of data gathered during the VA interview, was about twice as high among community deaths compared with hospital deaths.

While CSMFs comparisons of individual diseases or disease groups is of critical importance for planning, analysis by broad disease cause categories can help assess the extent to which a country has progressed through the epidemiological transition from communicable diseases and maternal causes of death, to NCDs. The relative fraction of deaths in each of the three broad cause categories defined by the GBD study (before-redistribution and excluding undetermined deaths) is shown in Table 4 based on the VA data. The data confirm that the epidemiological transition is well underway in the Solomon Islands, with over 70% of adult deaths due to NCDs. NCDs were even more common as causes of death for community VA deaths, but were much less common among hospital-related VA deaths, given the chronic nature of these conditions, although NCDs were still the dominant cause category for hospital deaths.

**Table 4:** Broad cause distribution (%) of VA deaths, by source, Solomon Islands, 2016–20

Broad cause (GBD Level I) group	Hospital related VAs (*n* = 91)	Community VAs (*n* = 819)	Total VAs (*n* = 914^a^)	GBD estimates (%)
Group I (communicable diseases, nutritional and maternal disorders)	25	14	15	16
Group II (non-communicable diseases)	57	75	73	71
Group III (injuries)	18	11	12	13

^a^Includes four deaths where location of death was not specified

**Fig. 2:**
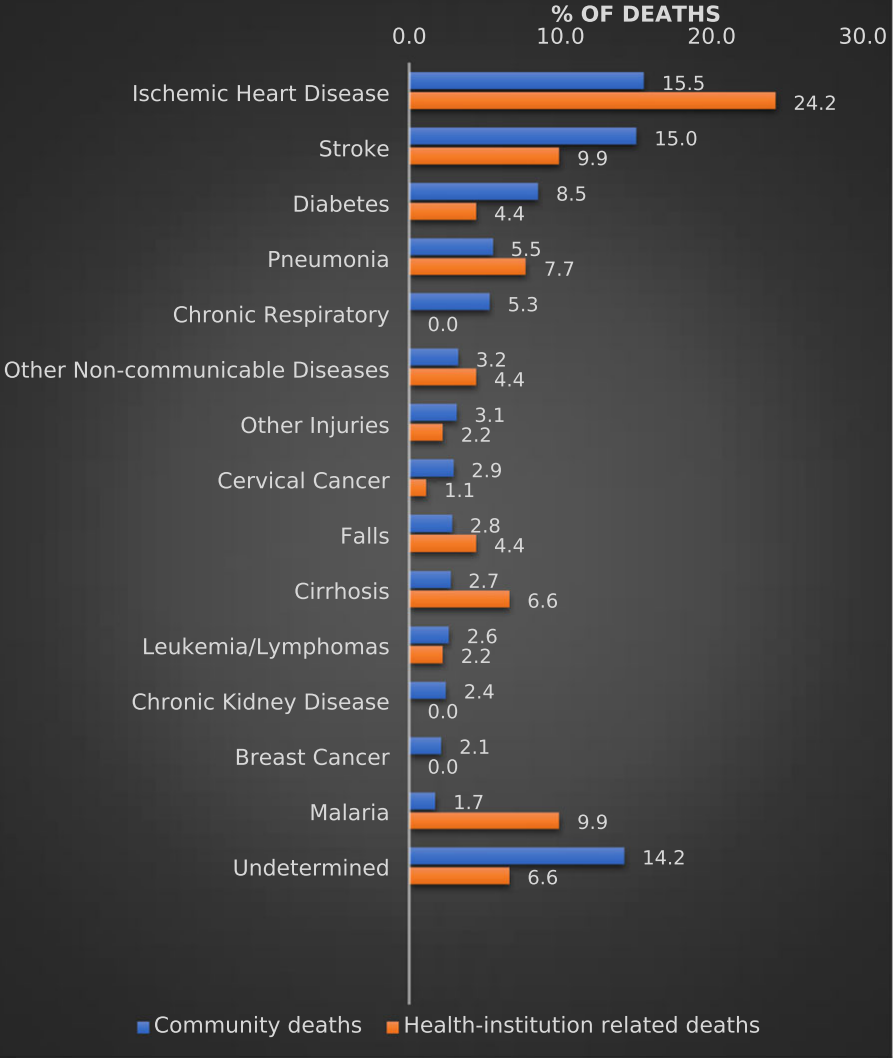
CSMFs for leading causes of death from VA data compared with GBD estimates, adult males, Solomon Islands, 2016–20

**Fig. 3:**
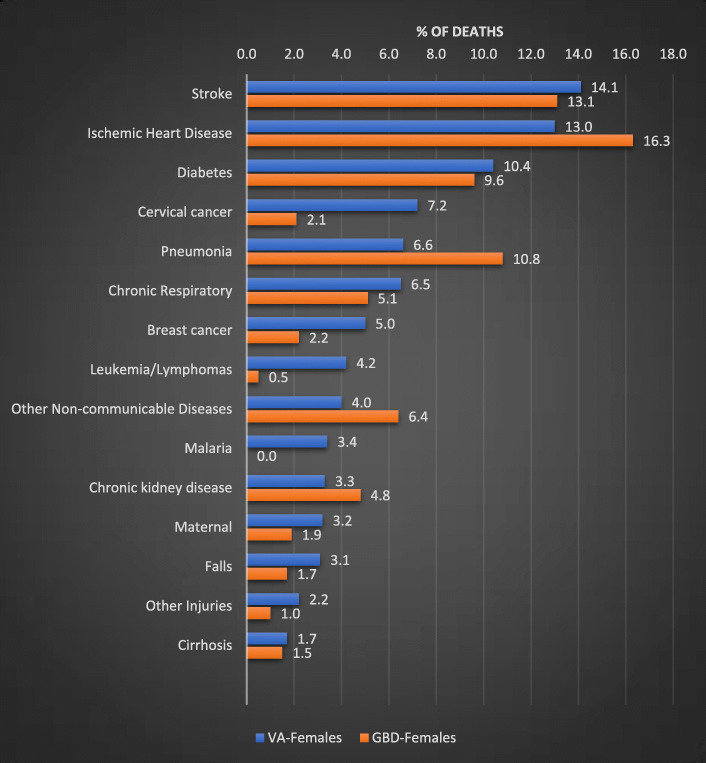
CSMFs for leading causes of death from VA data compared with GBD estimates, adult females, Solomon Islands, 2016–20

Figures 2 and 3 compare the CSMFs for adult VAs (after redistribution) with estimated national CSMFs from the GBD study, for males and females respectively. The cause pattern revealed by VA is remarkably similar to the GBD estimates, despite the very different estimation/data collection approaches. Among males, the VA data found that ‘other injuries’ (primarily accidental injuries) and liver cirrhosis were more common as a cause of death than suggested by the GBD Study, whereas pneumonia and road traffic accidents were less prominent than suggested by the GBD estimates. Among females, VA identified more breast and cervical cancer, and less ischaemic heart disease, pneumonia and ‘other non-communicable diseases’ than the GBD suggested was the case. For both sexes, stroke, malaria and leukemia/lymphoma were more common as a cause of death in the VA data compared to the indirect GBD estimates.

As part of a comprehensive program to improve mortality data in the Solomon Islands, there have been ongoing efforts to improve the diagnostic accuracy of physician certification for hospital deaths. For various reasons, one might expect the cause pattern of deaths in hospitals (subject to medical certification of cause of death, MCCOD) to differ from that in the community (assessed by VA). Table 5 presents the CSMFs from all adult VAs compared with the MCCOD data for adults who died in hospitals. While the male to female ratio of deaths subject to MCCOD was similar to the VA data (3:2), the median age at death of those receiving an MCCOD was 50 years, substantially younger than for VA deaths (62 years). This difference in age at death between the two data sources is reflected in the cause composition; of the ten leading causes among the MCCOD deaths, only six were also among the ten leading causes diagnosed by VA. Notably, diarrhoea and some cancers were more common among hospital deaths than the VA data (not present in the VA leading causes), whereas ischaemic heart disease, stroke and chronic respiratory disease, which tend to be more common among older adults, were identified as leading causes of community deaths as diagnosed from VA.

**Table 5:** Leading causes of death diagnosed by VA (ages 12+) compared with medically certified cause of death data (ages 15+), Solomon Islands, 2016–20^a^

	VA (*N* = 914)		MCCOD (*N* = 1408)	
	Cause	%	Cause	%
1	Ischemic heart disease	17.6	Other NCDs^b^	14.1
2	Stroke	15.7	Other infectious^c^	13.1
3	Diabetes	8.9	Ischemic heart disease	11.4
4	Pneumonia	6,5	Diabetes	11.2
5	Chronic respiratory disease	5.6	Stroke	8.7
6	Other NCDs^b^	4,3	Other cancers	6.5
7	Other injuries^d^	3.5	Other cardiovascular disorders	6.5
8	Cirrhosis	3.4	Leukemia/lymphoma	3.6
9	Malaria	3.3	Diarrhea/ dysentery	3.1
10	Falls	3.1	Chronic respiratory disease	2.7

^a^The related ICD codes are shown in supplementary Table 3

^b^NCDs which are not included within another specific cause-category of the SmartVA cause list

^c^Infectious conditions which are not included within another specific cause-category of the SmartVA cause list

^d^Injuries which are not included within another specific cause-category of the SmartVA cause list

## Discussion

To our knowledge, this study provides the first ever direct evidence about cause of death patterns in the Solomon Islands, based on verbal autopsy diagnoses for deaths which occur outside of health facilities, which are the vast majority of deaths in the country. At a broad cause level, about three out of four adult deaths are due to NCDs, one in six from a communicable, maternal or nutritional condition, and about 12% from injuries. The similarity of our findings with the modelled GBD estimates of national deaths, based on covariates and data from neighbouring countries, suggests that the national VA system now operating in the country is producing a plausible assessment of the relative importance of communicable vs non-communicable diseases, and injuries, as causes of community deaths, at least for adults. Furthermore, the VA data align reasonably closely with the GBD estimates in identifying the leading specific causes of community deaths among adults. Our findings also suggest that the age distribution and leading causes of community deaths as assessed by VA differ in important ways from medically certified hospital deaths (MCCOD deaths), reflecting the availability and likely impact of treatment.

The lower median age and more skewed male to female ratio among the hospital-related VA and MCCOD data, compared to that of community VA data, suggests that there are significant biases in using health facility data on causes of death to infer national mortality patterns, with younger adults and males more likely to die in or near a facility. These age and sex differences are also likely to account for much of the difference in COD patterns between facility and community deaths. Community VA sex ratios and median age at death were the closest to national estimates, and thus, are more likely to be a demographically representative sample than facility related deaths [7]. Nonetheless, the skewed sex ratio, even among community deaths, likely reflects a sampling bias in the VA system. While the VA system covers every health zone in every province, data collectors are not located in every community and are often responsible for large catchment areas requiring significant travel and communication constraints. It is probable that deaths which occur closer to Area Health Centres, and those voluntarily reported by communities to nurses, are the most likely to receive VAs. This may affect the generalisability of the results. To ensure that VAs are completed for a more representative sample of the population, efforts are underway to increase death notifications to nurses by strengthening community partnerships with churches, nurse aides, and cemetery officials, who collectively are aware of most deaths that occur in the community. The under-reporting of deaths is not uncommon in the Solomon Islands; the most recent national Census also found a skewed sex ratio in reported deaths and inferred a substantial underreporting of deaths at the household level [7].

The age distribution of adult deaths in the VA data was broadly congruent with GBD estimates of overall mortality, suggesting there was relatively little age-related sampling bias. The distribution of median age at death of adults was relatively high compared to some Pacific Island populations such as Papua New Guinea, but lower than for other low- to middle-income populations for which VA statistics are available, reflecting the better levels of overall health development in the country compared to Papua New Guinea in particular [14]. The reported higher median age at death of females compared to males is also consistent with the longer life expectancy for females in similar island nations in the region, as indeed for almost all other countries [22].

The leading five causes of death among men and women included communicable (pneumonia) as well as non-communicable diseases (ischemic heart disease, stroke, diabetes, cervical cancer and chronic respiratory disease), consistent with a country partway along the epidemiological transition. This transition was noted to be well underway in most Pacific island countries in the 2010 GBD study, [23] and may now be accelerating in Solomon Islands. There is evidence that both predisposing diseases and risk factors for NCD deaths are prevalent in Solomon Islands; for example, diabetes prevalence in Solomon Islands has been found to be as high as 17.8% among men and 14.3% among women aged 25 to 64 years, while worryingly high levels of key NCD risk factors such as heavy drinking (77.4% among men, 37.3% among women), smoking (52.6% in men, 24.3% in women), physical inactivity (37.6% in men, 47.4% in women) and overweight/obesity (63.8% among men, 72.3% among women) have been documented [24]. This, coupled with the decline in many communicable diseases due to the success of key disease control programs, including malaria control, vaccination programs and efforts to control diarrhoeal diseases and tuberculosis, has led to the dominance of NCDs as the leading cause of death in the country, contributing to the rapidly advancing epidemiological transition that our findings have documented [8].

Deaths due to falls, drowning and other injuries featured prominently among the leading 15 causes of death in the VA data. This reflects the broad challenges that the Solomon Islands faces in occupational and transport safety, where many people are engaged in private agriculture or fishing with little enforcement of safety standards, and travel is frequently by small watercraft with few safety features.

The important differences we observed between community mortality patterns, as diagnosed by VA, and physician-certified hospital deaths, demonstrates the need for policy and planning to rely on both for informed decision making and to provide a complete national picture of mortality. The leading causes of death in hospitals included a number of relatively unusual communicable and NCDs, typical of referral centres which offer more specialist care and therefore attract more complex or unusual cases. While ischaemic heart disease, stroke and diabetes were present as causes of hospital deaths, they were not as prominent as might be expected, even in hospital data. Overall, the hospital deaths data are not likely to be representative of mortality patterns in the population as a whole, and should not be relied upon alone to guide population-level health planning. The VA data presented a more plausible pattern of mortality for the whole population and thereby are likely to have more policy relevance in guiding programs designed to reduce premature death in the community.

In addition to demonstrating the utility of VA methods for identifying the leading causes of community deaths, typically the vast majority of deaths in LMICs, our study provides further support to the limited global evidence about the usefulness of VAs for diagnosing deaths on/by arrival at hospitals [25]. These are a unique set of deaths, being demographically and epidemiologically distinct from community deaths at home, and possibly different in access to transport and health facilities. Determining the cause of death of ‘dead on arrival’ cases is generally ignored given the sparse information usually available on these deaths, yet in some countries, they may constitute a significant fraction of hospital deaths. As our study has demonstrated, verbal autopsy can be used with reasonable success to ascertain the cause of death of these cases, thus filling an important information gap in the planning of health services. Further, understanding the characteristics of this group is important for addressing current challenges with referrals and patient transport. Similarly, gaining a more detailed and reliable understanding of mortality patterns in the community can inform outreach, health promotion and primary care services. SmartVA was able to provide critical health intelligence for both community deaths and dead on arrival cases with sufficient specificity to render the information useful for planning, as evidenced by the reasonably low (15%) ‘undetermined’ fraction, even for community deaths [1]. The Tariff 2.0 algorithm used by SmartVA is one of three algorithms commonly used for the automated analysis of VA data, the others being InterVA and InSilicoVA. While each have quite different methodologies, comparison studies have suggested that they yield broadly similar results [26, 27]. While it is likely that the findings reported in this study were affected by the choice of diagnostic algorithm, a comparative analysis with other algorithms to explore the pattern and extent of these differences is beyond the scope of our study. The Tariff diagnostic method was preferred since it performs at least as well as other algorithms and requires a shorter questionnaire.

This study has several limitations, including the low (but expanding) coverage of community deaths by VA, with a significant male death reporting bias, as well as a likely location sampling bias (reporting more deaths which occur close to facilities with a VA data collector). As a result, deaths in the more remote and rural settings may be under-represented, with possible implications for generalising the cause of death patterns we uncovered. The relatively small adult sample means that while conclusions regarding broad cause categories and the epidemiological transition are likely to be more robust, there is less precision regarding individual CSMFs. The very small samples of neonatal and child deaths did not allow for calculation of CSMFs. The higher fraction of deaths reported with cause ‘undetermined’ in community deaths compared to hospital deaths on/by arrival may be partly due to the observed demographic and epidemiological differences in the two cohorts, and the higher levels of clinical training and access to testing among hospital-based interviewers; but subsequent data quality audits have also identified concerns with interview technique among some community interviewers, and limitations in communication (for example, VA interviews conducted over radio or telephone, or with a second-hand informant). We have introduced several subsequent interventions, including refresher training, further supportive supervision, and community reporting mechanisms which seek to improve the quality of VA data for community deaths. While this first analysis of VA data must be interpreted with caution, it nonetheless represents, we believe, a substantial advance on current knowledge and practice based almost exclusively on hospital deaths. Further refinement of the VA methodology in the Solomon Islands, particularly ongoing initiatives to increase the coverage of VA to more remote communities and improve the quality of data collection, will ensure that decision makers and development partners have access to fundamental and reliable information about the leading causes of death, and how they are changing, in the predominantly rural population where most deaths occur.

## Conclusions

Non-communicable diseases were the predominant causes of deaths identified by verbal autopsy, indicating that Solomon Islands is well along the pathway of epidemiological transition. The automated VA methodology provided critically important health intelligence on the leading causes of adult deaths in the country and are likely to be more representative of population level mortality conditions in the country than the cause of death patterns derived from hospital (physician certified) data. In a setting such as Solomon Islands with limited access to physicians and MCCOD, automated VA is an effective method for generating policy-relevant CSMFs representative of non-inpatient deaths, and has considerable policy utility for health sector planning if it can be scaled and incorporated into the national routine mortality surveillance system.

## Supplementary Information

**Additional file 1.**

## Data Availability

The datasets used and/or analyzed during the current study are available from the Government of Solomon Islands on the recommendation from the National Mortality Technical Working Group on reasonable request.

## Abbreviations

AHC: Area Health Centre
COD: Cause of death
CRVS: Civil registration and vital statistics
CSMF: Cause specific mortality fraction
GBD: Global Burden of Disease
MCCOD: Medical certification of cause of death
NCD: Non-communicable disease
NMTWG: National Mortality Technical Working Group
ODK: Open Data Kit
VA: Verbal autopsy
