# Supplementary material for: Generating cause of death information to inform health policy: implementation of an automated verbal autopsy system in the Solomon Islands
Source: BMC Public Health. 2021 Nov 13;21:2080. doi: 10.1186/s12889-021-12180-y (PMC8590305; doi:10.1186/s12889-021-12180-y)
Supplement: Supplementary file 1 — Additional file 1. [file 12889_2021_12180_MOESM1_ESM.docx]

**Supplementary materials for “Generating cause of death information to inform health policy: application of automated verbal autopsy methods in the Solomon Islands”**

**Supplementary table 1: Mapping of ICD-10 codes to SmartVA cause categories**


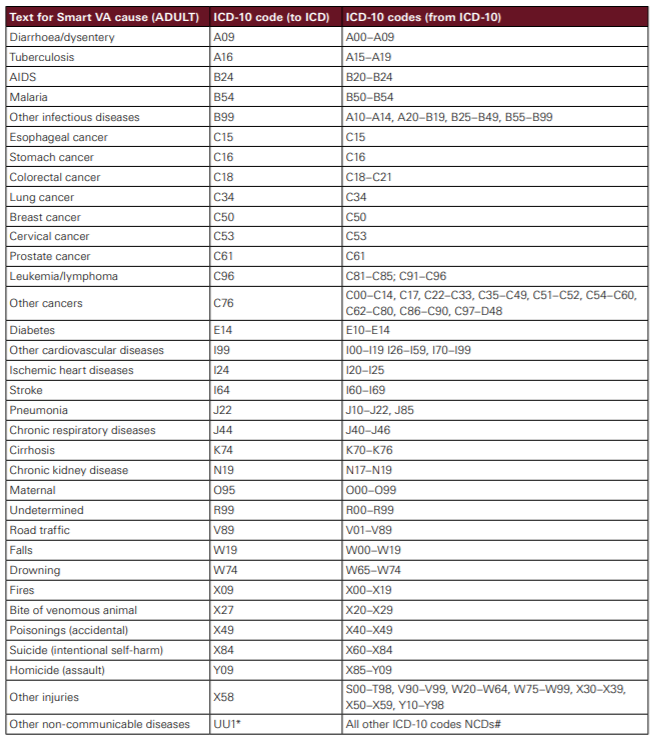


Source: VA interpretation guidelines (22)

**Supplementary table 2: Comparison of age distributions of VAs and GBD estimates**

| **Age groups** | **Both sexes** | | **Males** | | **Females** | |
| --- | --- | --- | --- | --- | --- | --- |
|  | **VA %** | **GBD %** | **VA %** | **GBD %** | **VA %** | **GBD %** |
| 10 to 14 | 1.2 | 2.1 | 1.4 | 2.3 | 0.8 | 1.9 |
| 15 to 19 | 1.9 | 3.3 | 2.3 | 3.0 | 1.1 | 3.6 |
| 20 to 24 | 2.5 | 4.3 | 2.5 | 3.1 | 2.5 | 5.6 |
| 25 to 29 | 3.3 | 4.8 | 3.6 | 3.9 | 2.8 | 5.8 |
| 30 to 34 | 4.9 | 5.9 | 4.6 | 4.7 | 5.4 | 7.2 |
| 35 to 39 | 2.8 | 7.0 | 2.5 | 5.7 | 3.4 | 8.4 |
| 40 to 44 | 5.4 | 6.2 | 5.9 | 6.6 | 4.5 | 5.8 |
| 45 to 49 | 5.3 | 5.8 | 5.5 | 6.3 | 4.8 | 5.2 |
| 50 to 54 | 8.2 | 6.5 | 8.9 | 6.8 | 7.1 | 6.1 |
| 55 to 59 | 6.6 | 6.6 | 8.2 | 7.4 | 4.0 | 5.7 |
| 60 to 64 | 9.7 | 7.4 | 9.5 | 8.8 | 10.2 | 5.9 |
| 65 to 69 | 6.8 | 8.1 | 5.9 | 9.9 | 8.2 | 6.2 |
| 70 to 74 | 10.7 | 8.5 | 9.6 | 9.1 | 12.4 | 7.7 |
| 75 to 79 | 7.8 | 9.0 | 7.0 | 9.0 | 9.0 | 9.0 |
| 80 Plus | 17.7 | 14.6 | 15.9 | 13.3 | 20.6 | 16.1 |
| Unknown | 5.3 | N/A | 6.6 | N/A | 3.1 | N/A |
| Total | 100.0 | 100.0 | 100.0 | 100.0 | 100.0 | 100.0 |

**Supplementary table 3: Leading VA causes by province**

| **Rank** | **Province** | | | | | |
| --- | --- | --- | --- | --- | --- | --- |
|  | **Guadalcanal+**  **Honiara** | **%** | **Western** | **%** | **Other** | **%** |
| 1 | Ischemic Heart Disease | 17.4 | Ischemic Heart Disease | 20.8 | Stroke | 14.9 |
| 2 | Stroke | 13.0 | Stroke | 15.7 | Ischemic Heart Disease | 12.5 |
| 3 | Pneumonia | 7.2 | Diabetes | 8.5 | Diabetes | 9.7 |
| 4 | Diabetes | 5.8 | Pneumonia | 4.2 | Chronic Respiratory | 5.5 |
| 5 | Malaria | 4.4 | Chronic Respiratory | 4.7 | Pneumonia | 5.5 |
| 6 | Other Injuries | 4.1 | Other Non-communicable Diseases | 3.8 | Cirrhosis | 4.4 |
| 7 | Chronic Respiratory | 4.1 | Other Injuries | 3.0 | Cervical Cancer | 4.2 |
| 8 | Leukemia/ Lymphomas | 3.4 | Falls | 3.0 | Falls | 3.1 |
| 9 | Other Non-communicable Diseases | 2.7 | Cervical Cancer | 3.0 | Other Non-communicable Diseases | 3.1 |
| 10 | Road Traffic | 2.7 | Leukemia/Lymphomas | 2.5 | Chronic Kidney Disease | 2.9 |
| 11 | Falls | 2.7 | Drowning | 2.1 | Other injuries | 2.3 |
| 12 | Chronic Kidney Disease | 2.4 | Breast Cancer | 2.1 | Malaria | 2.1 |
| 13 | Maternal | 2.0 | Cirrhosis | 1.7 | Leukemia/ Lymphomas | 1.8 |
| 14 | Esophageal cancer, lung cancer | 2.0 | Prostate Cancer, TB | 1.7 | Breast Cancer | 1.8 |
| 15 | Undetermined | 10.6 | Undetermined | 13.1 | Undetermined | 15.9 |
